# Off-trial evaluation of bisphosphonates in patients with metastatic breast cancer

**DOI:** 10.1186/1471-2407-5-89

**Published:** 2005-07-28

**Authors:** Winston Liauw, Eva Segelov, Anna Lih, Ms Ruth Dunleavy, Matthew Links, Robyn Ward

**Affiliations:** 1 Clinical Trials Centre, St Vincent's Hospital, Sydney, Australia; 2 Cancer Care Centre, St George Hospital, Sydney, Australia; 3 Department of Medical Oncology, St Vincent's Hospital, Sydney, Australia; 4 Concord Hospital, Concord. Sydney, Australia; 5 Faculty of Medicine, University of New South Wales, Sydney, Australia

## Abstract

**Background:**

Bisphosphonate therapy has been readily accepted as standard of care for individuals with bone metastases from breast cancer. In this study we determined whether the proportion of patients experiencing a skeletal related event (SRE) in a clinical practice population was similar to that observed in phase III randomized controlled studies.

**Methods:**

A retrospective chart review was conducted of 110 patients receiving intravenous bisphosphonates for advanced breast cancer. The proportion of patients experiencing at least one SRE after 12 months of therapy was determined. SRE included vertebral or non-vertebral fracture, cord compression, surgery and/or radiotherapy to bone.

**Results:**

The proportion of patients who had an SRE was 30% (28 individuals) and the median time to first event was greater than 350 days. Non-vertebral events and radiotherapy were the most frequent type of SRE, while cord compression and hypercalcaemia were rare (1%). Most patients in the study had bone-only disease (58.2%) and most had multiple bone lesions. In the first 12 months the mean duration of exposure to intravenous bisphosphonates was 261 days and most patients remained on treatment until just before death (median 27 days).

**Conclusion:**

This study suggests that the rate of clinically relevant SREs is substantially lower than the event rate observed in phase III clinical trials. We attribute this lower rate to observational bias. In the clinical trial setting it is possible that over-detection of skeletal events occurs due to the utilisation of regular skeletal survey or radionucleotide bone scan, whereas these procedures are not routine in clinical practice. Phase IV observational studies need to be conducted to determine the true benefits of bisphosphonate therapy in order to implement rationale use of bisphosphonates.

## Background

Randomised controlled studies have consistently demonstrated that the skeletal complications of metastatic breast cancer can be reduced by the regular administration of intravenous bisphosphonates [1-7]. Given the potential impact of a pathological fracture, it is not surprising that bisphosphonate therapy has been readily accepted as standard of care for metastatic bone disease [8-10]. However, this therapy is expensive, and since bisphosphonates have no impact on survival, their cost-effectiveness is primarily justified by the avoidance of radiotherapy or surgery [11,12].

To date, clinical trials of bisphosphonate therapy have failed to determine the optimal frequency of administration, timing of initiation or duration of use [9]. In practice, patients are treated on a three to four weekly basis for an indefinite period. Until their death, individuals with advanced cancer are therefore exposed to the risk of infusion-related adverse events, the possibility of nephrotoxicity, and the inconvenience of intravenous treatment. In an effort to promote rational prescribing of bisphosphonates, a number of medical and funding agencies have developed treatment guidelines which include rules for initiation and cessation of treatment [9,10]. Unfortunately, recent studies have demonstrated that adherence to these guidelines is universally poor [10,13,14]. We propose that prescribing habits are unlikely to change without evidence of the efficacy, impact on quality of life, and cost-effectiveness of bisphosphonates in routine clinical practice.

To the knowledge of the investigators, there has been no report of the outcomes of patients receiving bisphosphonates outside the conduct of a clinical trial. Given the impact of inappropriate prescribing, it seems unreasonable to assume that the benefits observed in randomized controlled studies will be perfectly replicated in a clinical practice population. Furthermore, individuals in the key randomized studies underwent regular skeletal surveys, and the skeletal-related events (SRE) identified in these trials represented the composite of clinically relevant as well as asymptomatic radiological changes [1-6]. Skeletal surveys are not used routinely in clinical practice, and thus only clinically relevant skeletal events are likely to be identified in this setting. In this study, we report the results of an audit of intravenous bisphosphonate use in patients with metastatic breast cancer in the setting of routine clinical practice.

## Methods

### Study cohort

110 women with metastatic breast cancer were included in this study. They had commenced intravenous pamidronate or zoledronic acid for the prophylaxis of skeletal complications between January 1998 and September 2003. Patients were excluded if they were receiving bisphosphonates therapy for osteoporosis or for the management of tumour-related hypercalcaemia. Individuals were identified through the pharmacy records of two large comprehensive cancer centers in Australia; St Vincent's Hospital, Darlinghurst, NSW, and St George Hospital, Kogarah, NSW. The dataset was extracted from medical and pharmacy records by three investigators (AL, RD, ES) and entered into a standardised data collection form. A random sample of forms was audited by an independent investigator (WL) and data queries were resolved by case review by three investigators (WL, ES, RW). Relevant clinical information as well as the details of bisphosphonates usage was recorded on each person for a period of 12 months from the time of commencement of bisphosphonate therapy.

The primary outcome measure used in this study was the proportion of patients experiencing at least one SRE in a 12 month period following the commencement of intravenous bisphosphonate therapy. SRE were defined as either a pathological fracture, a bony lesion requiring intervention (surgery or radiotherapy) for pain or prevention of skeletal complications, spinal cord compression or hypercalcaemia of malignancy. The total number of SRE did not include events occurring within 30 days of commencement of intravenous bisphosphonate therapy if they were related to the index presentation of bony disease. Simultaneous SRE (i.e. presenting on the same day) were counted as one event. Treatment of a single lesion with radiotherapy and surgery were not coded as separate SREs.

The study was conducted under guidelines consistent with the NHMRC National Statement of Ethical Conduct in Research Involving Humans.

### Statistical analysis

Descriptive statistics were used to define the characteristics of the sample and Kaplan-Meier (KM) estimates were used to calculate the proportion with an SRE. The KM technique was used to estimate the time from commencement of bisphosphonate administration to the first SRE. Only SRE not cancer-related deaths were analysed as events. All data was analysed using SPSS statistical software V11.0 (SPSS Inc., Chicago, IL).

## Results

### Patient characteristics and bisphosphonate administration

The baseline clinical characteristics of the study cohort are shown in Table 1. At initiation of bisphosphonates therapy the mean age of the group was 57.2 ± 12.1 years and most patients (50%) had multiple bone metastases. On average, skeletal disease had developed 4.2 ± 4.4 years from the primary diagnosis of breast cancer and bisphosphonate therapy was commenced at a mean of 291 ± 497 days from the time of diagnosis of bone metastases. Bisphosphonates were commenced concurrently with chemotherapy (either alone or with hormone therapy) in 54.4% of patients, with hormonal therapy in 40.4% of patients, and without any systemic treatment in 5.3% of individuals. In the first 12 months the mean duration of exposure to intravenous bisphosphonates was 261 days, and a total of 60 patients (54.5%) remained on therapy beyond the 12 month observation period. The mean and median number of infusions of intravenous bisphosphonates in the first 12 months of follow-up was 9 and 12 respectively. Over the total duration of follow-up a mean of 16 cycles of IV bisphosphonate were received, with an upper range of 56 cycles. The reasons for discontinuation of bisphosphonates within the 12 month observation period were cancer death (24 patients, 22%); change to oral bisphosphonates (13 patients, 12%); intolerance (5 patients, 4.5%) or not specified (3 patients, 2.7%). A significant number of patients remained on therapy despite being close to death from cancer progression. In the 24 patients who died within the study period, the time from last documented bisphosphonate infusion to death was between 2 and 140 days with a median of 27 days. Although bisphosphonate treatment guidelines state that an abnormal bone scan without evidence of bone destruction does not justify the initiation of bisphosphonates, we found that 21% (23 individuals) of individuals in this audit had commenced treatment on this basis.

### Skeletal complications

**Figure 1:**
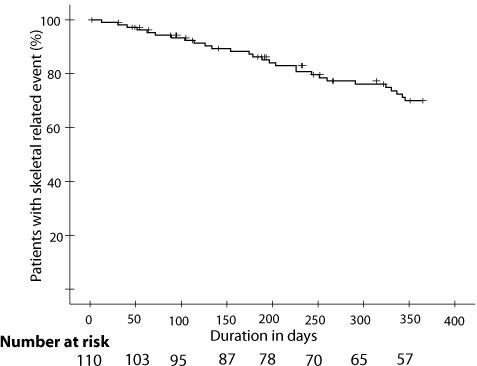
Kaplan-Meier estimates of the time to first skeletal related event from the date of first intravenous bisphosphonate infusion. Censored at 12 months follow-up.

The proportion of patients experiencing at least one SRE within 12 months of commencing bisphosphonates was 30% (28 individuals). The characteristics of these events, and the proportion of patients experiencing them, are described in Table 2. Spinal cord compression and hypercalcaemia were rare events, occurring in only 1% of individuals within the study period. The median time to the first occurrence of a SRE was greater than 365 days (Figure 1). Within the 12 month observation period, nine of 28 individuals developed a second SRE at a mean of 223 days from bisphosphonate initiation, and of these six were still undergoing treatment with bisphosphonate at the time of the second SRE. Three individuals developed a fourth SRE and one a fifth SRE within 12 months of starting bisphosphonates.

Interestingly, when patients with bone scan only disease were excluded from the analysis the cumulative proportion of individuals developing a SRE within the first year of commencing bisphosphonates was 32% (23 of 87 patients). There was no statistically significant difference between subjects with bone scan-only detected metastases and the remainder of the cohort in the proportion of subjects with an SRE in the first 12 months (p = 0.77). This data suggests that bone scan only disease does not represent a better prognostic group of patients.

## Discussion

Over 80% of women with metastatic breast cancer have bone metastases [15,16], yet a much smaller proportion of these will develop clinically apparent complications related to bone destruction. One large retrospective study performed before the introduction of bisphosphonates demonstrated that 29% of individuals develop a clinically significant SRE [15]. This figure is comparable to the findings of the current study (30%) yet different from the results of the randomized controlled trials of intravenous bisphosphonates [1-6]. In these latter studies, the proportion of individuals who experienced at least one skeletal complication at 12 months from bisphosphonate commencement was 43% in the treatment arm compared with 56% in the placebo arm. We propose that the discrepancy between the results of the randomized controlled studies and our current audit relates primarily to the use of regular radiographic skeletal surveys in the trial setting [4]. In practice, skeletal events are only identified on the basis of clinical suspicion and thus many bone lesions may be appropriately undetected and untreated. The corollary is of course that intensive exposure to skeletal surveys may result in the treatment of lesions which may never become clinically significant.

The baseline population risk of a skeletal event is a further factor which may explain the discrepancies between the current audit and the results of previous randomized controlled studies. Patients with metastatic disease limited to bone are four times more likely to fracture a long bone than those with concurrent liver and bony metastases, while individuals with extensive metastasis involving long bones are more likely to develop a fracture than those with solitary metastases [17]. In terms of these risk factors, our patient population was certainly at no lesser risk of fracture than the individuals enrolled in the studies of Hortobagyi et al [1,2] and Theriault [3]. The percentage of individuals with metastatic disease limited to bone was comparable at 58.2% (current audit), 60% (Hortobagyi studies) and 70% (Theriault et al,). Furthermore, only 9.1% of the subjects in the current study had a solitary bone metastasis, whereas 43% of patients in the Hortobagyi studies had an isolated lesion [1,2].

The endpoint used in this study, specifically the proportion of patients with more than one SRE is well accepted and provides readily assessable and comparable estimations of treatment effect. It however only captures information about the first event and does not measure the multiple skeletal events which sometimes occur in a single individual. While acknowledging that the impact of bisphosphonate therapy on recurrent events is important the best way to measure and analyse this data is controversial [18-21]. For this reason, we chose not to evaluate secondary endpoints such as total number of SRE, and event rate in the current audit.

Given the relative risk reduction in skeletal related events bisphosphonates clearly have a place in the management of women with metastatic bone disease. It is uncertain however what the absolute benefits of these reductions are, whether in terms of SRE, or economic endpoints. The rationale use of these agents is hindered by a lack of data concerning drug scheduling, duration of use, indications for initiation and cessation [9]. This audit highlights the fact that while these questions remain unanswered, clinicians will be reluctant to alter current prescribing habits. Most patients remained on bisphosphonates despite a declining performance status; the drugs were indefinitely administered on a three-four weekly basis and 67% of patients who experienced a second SRE continued bisphosphonate therapy.

## Conclusion

The findings of this study provide an additional impetus to proceed with post-marketing evaluation of the use of bisphosphonates in clinical practice.

## Competing interests

RW is a member of the Pharmaceutical Benefits Advisory Committee (PBAC), Commonwealth Department of Health and Ageing, Canberra, ACT, Australia. The views presented here are those of the authors and should not be understood or quoted as being made on behalf of the PBAC or its Scientific Committees. The other authors declare that they have no competing interests.

## Authors' contributions

WL carried out statistical analysis, collected data and prepared draft versions of the manuscript. ES collected data and prepared draft versions of the manuscript. AL collected data. RD collected data. ML collected data and participated in the design of the study. RW conceived and coordinated the study, carried out statistical analysis and prepared the final versions of the manuscript. The final manuscript was approved by all authors.

## Pre-publication history

The pre-publication history for this paper can be accessed here:



## Figures and Tables

**Table 1:** Disease characteristics of patients

	Number of patients	Percent (%)
Sites of extra-osseous disease		
None	64	58.2
Liver Only	14	12.7
Brain Only	1	0.9
Lung Only	3	2.7
Multiple	11	10.0
Other	15	13.6
Unknown	2	1.8
Number of bone lesions		
1	10	9.1
2–5	40	36.4
>5	55	50.0
Unknown	5	4.5
Treatment prior to bisphosphonates		
Nil	33	30.0
Chemotherapy +/- hormone	31	28.2
Hormone therapy alone	46	41.8
Hormone therapy prior to bisphosphonates		
1^st ^line	31	28.2
2^nd ^line	19	17.3
3^rd ^line	3	2.7
Not applicable	57	51.8
Median survival from start of bisphosphonates	Days (95% CI)	
		
All subjects	818 (644–911)	
Subjects with bone metastases only	998 (653–1342)	
Subjects with extra-osseous metastases	513 (339–686)	

* based on available imaging, either bone scan, skeletal survey, CT or MRI.

**Table 2:** Proportion of patients having each type of SRE in the first 12 months of intravenous bisphosphonate therapy

	Number of patients	^¥^Proportion with event (%)
Type of SRE		
Pathological fracture	7	9%
Cord compression	1	1%
Other	20	23%
Site of SRE		
Vertebral	9	10%
Non-vertebral	17	22%
Hypercalcaemia	1	1%
Treatment for SRE		
Radiotherapy	17	21%
Surgery	3	4%
Medical^+^	8	10%

^¥^Kaplan-Meier estimates of the proportion with a complication. ^+ ^Medical includes change in analgesics, hormonal treatment or chemotherapy.
